# Genome Sequence of *Thermoanaerobaculum aquaticum* MP-01^T^, the First Cultivated Member of *Acidobacteria* Subdivision 23, Isolated from a Hot Spring

**DOI:** 10.1128/genomeA.00570-14

**Published:** 2014-06-12

**Authors:** Blake W. Stamps, Nathaniel A. Losey, Paul A. Lawson, Bradley S. Stevenson

**Affiliations:** Department of Microbiology and Plant Biology, University of Oklahoma, Norman, Oklahoma, USA

## Abstract

*Thermoanaerobaculum aquaticum* MP-01^T^ is currently the only cultivated and described member of *Acidobacteria* subdivision 23. Here, we report the genome sequence for this novel microorganism that was isolated from a hot spring.

## GENOME ANNOUNCEMENT

*Thermoanaerobaculum* is the first genus to be identified within *Acidobacteria* subdivision 23. Environmental sequences from members of the phylum *Acidobacteria* are found throughout a broad diversity of ecosystems, including soils, caves, hot springs, and deep sea hydrothermal vents (1–4). Based on 16S rRNA gene sequence divergence, the phylum *Acidobacteria* is predicted to be as diverse as the much better studied *Proteobacteria* (5). Among the 26 proposed subdivisions of *Acidobacteria* (1), only 6 (subdivisions 1, 3, 4, 8, 10, and 23) are represented by at least one cultivated member that has been described in detail. Currently, only 17 genome sequences are publicly available. Given the scarcity of data for the diverse members of the phylum *Acidobacteria*, it was imperative to sequence the genome of *Thermoanaerobaculum aquaticum* MP-01^T^, currently the only member of subdivision 23. *T. aquaticum* MP-01^T^ is a nonmotile, Gram-negative, rod-shaped bacterium. It is a strictly anaerobic chemoorganotroph isolated from Hale House Spring in Hot Springs National Park, Arkansas. The spring is covered, lacking any direct terrestrial input of carbon. The waters are oligotrophic, with the primary source of carbon input being carbonate (6). MP-01^T^ was found to be capable of reducing Fe(III) or Mn(IV) and was able to grow fermentatively on proteinaceous compounds (7).

Genomic DNA was isolated from MP-01^T^ cells using the phenol-chloroform method, as described previously (8). A draft genome sequence was determined from a 350-bp insert library prepared with the Illumina TruSeq LT kit and sequenced using the Illumina MiSeq instrument for 2 × 150 bp paired-end sequencing. Raw reads were trimmed within CLC Genomics Workbench 7.0 (CLC bio, Cambridge, MA) to remove adapters, bases below Q30 (Phred 33), and 10 bp from each end. The trimmed sequences (>100 bp) were assembled and scaffolded *de novo* within CLC, retaining scaffolds of >800 bp. The assembly produced 68 scaffolds, for a final assembly size of 2.66 Mbp. The *N*_50_ of the genome is 115.9 kbp, and the largest scaffold is 280.1 kbp.

The NCBI Prokaryotic Genome Annotation Pipeline was used for gene annotation (http://www.ncbi.nlm.nih.gov/genome/annotation_prok/). The genome contains 2,253 coding regions and 49 RNAs. Interestingly, while no autotrophic growth was observed in isolated cultures of MP-01^T^, genes required to fix carbon through reductive carboxylation are present, indicating that the potential may exist. During fermentation, MP-01^T^ was observed to produce hydrogen, butyrate, and lactate (7), which was confirmed by the presence of genes responsible for the metabolism of glucose, fructose, and pyruvate. No evidence for the ability to reduce sulfate or nitrate was found. Given the low relative abundance of MP-01^T^ observed in previous studies at Hale Springs (9), it is possible that MP-01^T^ subsists off the metabolic end products of other more dominant members of the community.

### Nucleotide sequence accession number.

This whole-genome shotgun project has been deposited in GenBank under the accession no. JMFG00000000.
